# Genetic Signature of Breast Cancer Defines Therapeutic Interventions and Indicates Early Drug Resistance

**DOI:** 10.1111/jcmm.70635

**Published:** 2025-05-28

**Authors:** Angeliki Margoni, Kostas A. Papavassiliou, Athanasios G. Papavassiliou

**Affiliations:** ^1^ Department of Biological Chemistry, Medical School National and Kapodistrian University of Athens Athens Greece; ^2^ First University Department of Respiratory Medicine, “Sotiria” Chest Hospital, Medical School National and Kapodistrian University of Athens Athens Greece

**Keywords:** breast cancer, drug resistance, genetic testing, precision oncology, targeted therapy

## Abstract

Breast cancer (BC) is the most prevalent cancer in women and ranks first in diagnosed cancers worldwide, thanks to effective screening strategies. However, morbidity and mortality rates remain high among aggressive subtypes, forcing oncologists to adopt the philosophy of precision oncology in clinical practice. Immunohistochemical analysis of the tumour establishes diagnosis and stratifies BC into four types, according to a globally recognised classification. Tumour cells present a wide diversity in morphological, histological and molecular characteristics, indicating the heterogeneous nature of BC that reflects on different proliferation rates, risks of relapse and metastasis. The differentiation in prognosis and response to treatment underscores the vital need for targeted therapy, specific for each tumour's genetic status, that enhances therapeutic effectiveness. The aim of the present article is to designate the potential of using a novel molecular modality as a diagnostic tool that enlightens oncologists during their therapeutic decisions and raises awareness of therapeutic failure. Next‐generation sequencing (NGS) detects a wide gamut of biomarkers and gene mutations in biopsy samples, revealing the precise genetic profile and implicating a personalised therapy. Incorporation of molecular testing in the diagnostic algorithm promises amelioration of therapeutic outcomes, prevention of recurrence and reduction of BC‐related mortality.

**Abbreviations**
ADC: antibody‐drug conjugates
AR: androgen receptor
BC: breast cancer
BRCA1/2: breast cancer gene 1/2
CD31/34: cluster of differentiation 31/34
CDK: cyclin‐dependent kinase
CK5/6: cytokeratins 5/6
CNB: core needle biopsy
DFP: disease‐free period
EGFR1: epidermal growth factor receptor type 1
ER: oestrogen receptor
FNAB: fine needle aspiration biopsy
HER2: human epidermal growth factor receptor 2
HT: hormone treatment
Ki67: antigen Kiel 67
MAPK: mitogen‐activated protein kinase
miRNAs: microRNAs
NGS: next‐generation sequencing
OC: ovarian cancer
OS: overall survival
OSR: overall survival rate
p53: tumour protein 53
PARPI: poly ADP‐ribose polymerase inhibitor
PR: progesterone receptor
PTEN: phosphate and tensin homologue
TKI: tyrosine kinase inhibitor
TNBC: triple negative breast cancer
VEGF: vascular endothelial growth factor

## 1 Introduction

Breast cancer (BC) is the most diagnosed cancer globally and the most frequent in women. The development of novel therapies has increased the overall survival rate (OSR) in BC. However, the mortality rate remains high in 20%–30% of BC subtypes that present increased aggressiveness and high risk of relapse [1, 2]. In recent years, ultrasonography, mammography and elastography have been well established worldwide as screening tests, partly explaining the rising incidence of BC in developed nations [2]. Nevertheless, unhealthy diet, sedentary way of living, smoking and alcohol consumption are inculpated for the increased rate in western societies, along with post‐menopausal hormone treatment (HT) and iatrogenic fertility patterns [2]. A thorough understanding of the causes and pathobiological pathways that lead to BC oncogenesis is challenging due to high diversity among different types of BC tumours [1, 3]. Therefore, the diagnostic algorithm should be enriched with the latest technology, molecular and genetic tests that offer a spherical perception of each type's features [4]. BC is a hereditary disease with a genetic, morphological and clinical heterogeneous nature that highlights the necessity for accurate tumour stratification to ensure effective treatment interventions [1, 2, 3, 4, 5, 6].

## 2 Classification of BC: Molecular and Genetic Diversity Prejudges Differentiation in Prognosis

The globally accepted classification of BC is based on immunohistochemical analysis that categorises BC in relation to the expression of oestrogen (ER), progesterone (PR) and human epidermal growth factor receptor 2 (HER2) receptors in BC cells [1, 2]. Accordingly, BC is stratified into four subtypes: Luminal A, Luminal B, HER2(+) and triple negative BC (TNBC) that present discrepancies in overall survival (OS) and disease‐free period (DFP) (Table 1). ER and PR are abundantly expressed in BC cells, supporting their diagnostic and prognostic significance as biomarkers [1, 2, 6]. ER(−) tumours are most frequent at younger ages and are correlated with higher mortality rates [3]. Epidemiologically, it is well established that higher expression of ER and PR is positively correlated with OS and time to recurrence, while lower expression is associated with poor prognosis [1, 3, 4]. HER2 is regarded as a proliferation marker, as it induces the rapid growth and spread of BC cells. In HER2(+) type, BC cells express excessive amounts of this growth factor compared to negative ones, but this carcinoma is likely to respond to anti‐HER2 drugs [6, 7, 8]. Overexpression of HER2 occurs at the beginning of oncogenesis, indicating that HER2 is a promising real‐time prognostic marker, as its levels are correlated with the recurrence of tumours and shortening of DFP [7, 8]. Antigen Kiel 67 (Ki67) is a biomarker that reflects aggressiveness and is related to lower survival rates [1, 4, 5, 6]. Ki67 is a protein that is found only in dividing cells and is expressed at higher levels in subtype Luminal B compared to Luminal A, partly explaining the difference in tumour progression between these two types (Table 1). A high proliferation index, also called K167‐score, is observed in subtypes with poor prognosis and claims to be an important marker for choosing an optimal individual therapy and an effective follow‐up strategy [1, 4, 5, 6, 8]. Additionally, cyclin‐dependent kinase 11 (CDK11) is a tumour suppressor kinase that hampers the role of vascular endothelial growth factor (VEGF), cluster of differentiation 31 (CD31) and CD34 in the growth and angiogenesis of tumour cells. Higher levels of VEGF are associated with poor prognosis, commonly in metastatic and TNBC tumours [3, 5, 8, 9]. Mutations in p53 are correlated with an increased risk of mortality and shortening of DFP independently of other risk factors [8]. Nowadays, molecular medicine and genomics unravel the complex and heterogeneous nature of BC by identifying several molecular markers such as microRNAs (miRNAs) (let‐7, miR‐155, miR‐153) and mutations in certain genes (*p53*, *breast cancer gene 1 and 2* [*BRCA1* and *BRCA2*]) [4, 5, 6, 9]. Mutations of BRCA1 and BRCA2 present a variable penetrance of inherited risk for BC; they are widely prevalent in familial BC but rarely detected in sporadic BC. Specifically, BRCA1 is the dominant mutation in familial BC and ovarian cancer (OC), whereas BRCA2 is less associated with OC but highly detected in familial male and female BC [8, 9]. Furthermore, BRCA1 interacts with p53 and ER, unveiling the role of BRCA proteins in sex steroid‐regulated pathways that induce BC. Mutations in the *BRCA1* gene are related to poor prognosis, low OS and higher recurrence than alterations in *BRCA2* [8, 9]. The morphological and molecular diversity among BC subtypes reflects a different response to therapy and a wide variability in prognosis. Tumour cells in the most common type, Luminal A, resemble normal breast cells. Luminal A is regarded as a slowly‐growing tumour with good prognosis, less incidence of relapse, that expresses primarily ER and PR and not HER2 (Table 1). Luminal A tumours present a high response to HT and more limited benefit to chemotherapy [1, 3]. Luminal B tumours have slightly poorer prognosis and tend to grow more rapidly than type A, partly due to high levels of Ki67. Luminal B tumours typically have ER but not PR and may or may not express HER2. These tumours may benefit from HT along with chemotherapy [1, 3, 7, 10] (Table 1). HER2(+) is a fast‐growing type that tends to be invasive and expresses high levels of HER2 with the absence of ER and PR. Although it has a worse prognosis than the luminal ones, research strongly supports that early diagnosis and combined specific anti‐HER2 targeted therapy, along with surgery and precise chemotherapy, increase the survival rate [1, 3, 7, 10] (Table 1). TNBC is an invasive type that tends to grow and spread quickly enough to be metastatic by the time of diagnosis. TNBC diagnosis is established when cells are negative for ER and PR, while they do not overexpress HER2, divulging that HT and anti‐HER2 drugs are ineffective for this tumour type [3, 8, 11, 12] (Table 1). It is characterised by a high proliferation rate, increased levels of Ki67 and genomic instability, whilst it constitutes 15%–20% of BC incidents and occurs commonly in younger women under the age of 40 (Table 1). TNBC displays excessive aggressiveness, increased risk of recurrence and low OSR. Histologically, it is a heterogenic and highly differentiated tumour, which is subdivided into basal and non‐basal type. Basal TNBC is characterised by the expression of cytokeratins (CK)5/6 and human epidermal growth factor receptor type 1 (EGFR1), whereas the non‐basal type does not express CK5/6 [8]. In TNBC, genetic profiling revealed alterations in cell cycle and DNA repair genes, growth factor receptor amplifications, mutations in phosphate and tensin homologue (PTEN) and mitogen‐activated protein kinase (MAPK) alterations [11, 12, 13]. This diversity in functional alterations enhances variability in drug sensitivity, DFP and prognosis among TNBC subsets. Nonetheless, it exhibits a higher response to neoadjuvant chemotherapy with platinum agents but fails to increase OSR in metastatic cases, known as the ‘TNBC paradox’ [11, 12, 13]. Novel strategies based on the genetic signature of the tumour lead to promising therapies with androgen receptor (AR)‐targeted drugs, antibody‐drug conjugates (ADC), poly ADP‐ribose polymerase inhibitors (PARPI), or tyrosine kinase inhibitors (TKI) [8, 11, 12, 13].

**TABLE 1:** Breast cancer classification based on cytological/molecular/genetic analysis in relation to prognosis and therapeutic strategies of choice.

	Luminal A	Luminal B	HER2(+)	TNBC
Prevalence (%)	50%	10%–20%	10%–15%	15%–20%
Prognosis	Good	Middle	Middle/Poor	Poor
Treatment of choice	Hormonal	Hormonal/Chemo	Hormonal/Chemo/Anti‐HER2	Chemo ADC/AR/TKI/PARPI
Rate of local/regional recurrence (%)	1.5/0.7%	2.9/1.5%	7.5/3.4%	7.6/3.3%
ER	(+)	(+)	(+/−)	(−)
PR	(+)	(+/−)	(+/−)	(−)
HER2	(−)	(−)	(+)	(−)
Ki67‐score	< 20% score	> 20% score < 30%	> 30% score	> 30% score
miRNAs	Let‐7f, Let‐7c, miR‐10, miR‐29a, miR‐181a, miR‐223, miR‐652	miR‐155, miR‐93, miR‐18a, miR‐718, miR‐4516, miR‐135b, miR‐210, miR‐125b‐5p	miR‐150, miR‐142‐3p	miR‐153, miR‐10b, miR‐26a, miR‐146a
Mutations	No	BRCA2	p53	p53 and BRCA1

Abbreviations: ADC, antibody‐drug conjugates; AR, androgen receptor; BRCA1/2, breast cancer gene 1/2; ER, oestrogen receptor; HER2, human epidermal growth factor receptor 2; Ki67, antigen Kiel 67; miRNAs, microRNAs; p53, tumour protein 53; PARPI, poly ADP‐ribose polymerase inhibitor; PR, progesterone receptor; TKI, tyrosine kinase inhibitor; TNBC, triple negative breast cancer.

## 3 Genomics and Molecular Medicine Guide Therapeutic Decisions

The widely used procedure after the detection of a breast nodule that raises clinical suspicion is core needle biopsy (CNB), as it has higher diagnostic accuracy and enables both molecular and immunohistochemical evaluation compared to the less invasive fine needle aspiration biopsy (FNAB) [14, 15, 16]. BC classification based on immunohistochemistry has limitations in terms of reliability, reproducibility and variability of laboratory techniques and pathologists' interpretations, requiring to be upgraded by employing the latest advances in molecular medicine [4, 14, 16]. As numerous molecular biomarkers, growth factors and gene mutations are implicated in BC progression, it is essential to be identified and co‐evaluated in advance of any therapeutic decision. The co‐existence of molecular biomarkers in a multiplex panel generates a diagnostic tool with high prognostic value, as early detection of the precise tumours' profile is inversely proportional to the BC mortality rate [5, 6]. The emerging gene expression profiling introduces patients to the benefits of precision oncology and ameliorates patients' adherence to treatment, as they embrace the potential of personalised therapy. A multi‐gene panel based on next‐generation sequencing (NGS) serves as an accurate prognostic and diagnostic tool that guides decisions in treatment protocols, relieving patients from ineffective interventions and unnecessary side effects [17, 18]. Although further clinical validation is warranted, a modality that defines the tumours' genetic identity should be integrated into the analysis of CNB samples. Automated next‐generation molecular testing based on NGS offers increased standardisation, precision and robustness in the detection of the carcinomas' features, while it promises a targeted therapy that decreases BC‐related morbidity and mortality [17, 19]. Oncologists anticipate to provide a breakthrough treatment strategy according to the molecular signature of the tumour, by integrating genetic profiling with BCs' diagnostic routine. Currently, the co‐knowledge of the molecular and histological nature of a BC tumour leads to specific therapeutic combinations in relation to subtypes (Table 1). Moreover, genomics facilitates the decision for the sort, the duration and the chronological sequence of combinatorial therapy, while it offers effective alternatives when toxic effects or drug resistance occur [17, 19].

## 4 Molecular Measurement of Biomarkers and Genes Expression Provides a Real‐Time Monitoring of Drug Resistance and BC Recurrence

Drug resistance to BC is a global burden that augments the risk of local or regional metastasis, early recurrence and poor prognosis. The role of genomics in real‐time monitoring of drugs' sensitivity is yet to be investigated. However, this article posits that a follow‐up sampling for early detection of gene mutations, alterations and increases in proliferation indexes (Ki67, HER2) is of utmost importance for the prevention of BC relapse or metastasis and improvement of prognosis, by timely detecting drug resistance [6, 10, 16]. Molecular techniques promise measurement of the pharmacodynamic effect of therapy, redirecting oncologists on time when proliferation biomarkers are elevated [1, 4, 5, 6, 13]. The establishment of a follow‐up algorithm based on genomics that monitors for increased biomarkers of recurrence or metastasis may restrain drug resistance and prevent therapeutic failure [5, 13, 17, 18, 19]. Patients who undergo extensive surgeries and combined therapies will endorse a post‐therapy genetic testing that assures their treatment effectiveness. Further clinical investigation is required, primarily to elucidate the role of a follow‐up multi‐test in increasing OSR, and secondly to define the appropriate sample for this procedure [14, 15, 16, 17, 19]. Besides, given the high cost of NGS technology, future studies that involve cost‐effectiveness are mandatory to proceed to the implementation of this algorithm in the clinical setting.

## 5 Conclusion

The rising incidence of BC in developed societies, along with the rather high mortality rate in aggressive subtypes, highlights the commitment of oncologists to utilise all latest technologies for social benefit. The evolution in molecular methods suggests an accurate and spherical diagnostic and therapeutic approach of BC. A diagnostic multi‐panel that combines several genes and biomarkers allows for unmasking the fully genetic and molecular nature of the tumour, while it promises an effective targeted regimen and an early indication of therapeutic failure.

## Author Contributions

**Angeliki Margoni:** conceptualization (lead), data curation (lead), writing – original draft (lead). **Kostas A. Papavassiliou:** conceptualization (equal), data curation (equal), writing – original draft (equal). **Athanasios G. Papavassiliou:** conceptualization (lead), data curation (lead), supervision (lead), writing – review and editing (lead).

## Disclosure

The authors have nothing to report.

## Conflicts of Interest

The authors declare no conflicts of interest.

## Data Availability

The authors have nothing to report.
